# Extracellular Vesicles in Health and Disease

**DOI:** 10.14336/AD.2021.0827

**Published:** 2021-09-01

**Authors:** Ashok K Shetty, Raghavendra Upadhya

**Affiliations:** Institute for Regenerative Medicine, Department of Molecular and Cellular Medicine, Texas A&M University College of Medicine, College Station, TX, USA

**Keywords:** Aging and senescence, Chronic traumatic encephalopathy, Extracellular vesicles as biomarkers, Extracellular vesicle therapy, Inflammatory bowel disease, Multiple sclerosis, PD, Sjogren’s syndrome

## Abstract

The journal, *Aging and Disease*, has released a special issue on “Extracellular Vesicles (EVs) in Health and Disease.” The special issue comprises review and original research articles discussing the role of EVs in aging and senescence, the utility of evaluating EVs in body fluids for understanding the pathophysiology or progression of various diseases such as Parkinson’s Disease, Multiple Sclerosis, Chronic Traumatic Encephalopathy, and Morphine induced amyloidopathy. Also, a series of articles discussed the promise of stem cell-derived EVs for treating Parkinson’s Disease, Sjogren’s Syndrome, and Inflammatory Bowel Disease, and advancements in loading EVs to deliver nucleic acid therapies. This editorial discusses the highlights from these articles.

Extracellular vesicles (EVs), nano-sized double membrane-enclosed vesicles shed by cells carrying a cargo of nucleic acids, proteins, lipids, and small molecules, are involved in intercellular communication [1,2]. The EVs, such as exosomes, come from the endosomal pathway, whereas microvesicles bud off directly from the plasma membrane. The markers of EVs comprise tetraspanins CD9, CD63, CD81, the endosomal protein tumor susceptibility gene 101, and ALG-2-interacting protein X [1-7]. The commonly found lipids in EVs comprise cholesterol, phosphatidylserine and phosphatidylinositol, sphingomyelin, and phosphatidyl-choline. Due to an overlap in their size range and marker expression, the categorization of EVs as either exosomes or microvesicles requires rigorous characterization of EV properties. Therefore, the International Society for Extracellular Vesicles (ISEV) has recommended employing operational terms in the absence of evidence supporting the release of exosomes by endosomes [8]. Hence, the phrase “EVs” is the most appropriate to describe both small (30-150 nm) and larger vesicles (100-1000 nm) shed by cells.

EVs can pass through multiple barriers in the organism, including the blood-brain barrier in the central nervous system (CNS) [9-10]. Because of their easy mobility, EVs play crucial roles in transferring genetic information to cells in both healthy and disease states. For example, in physiological conditions, EVs can influence the function of cells in distant places from the site of origin [11-12]. The EVs also have a role in the development, tissue repair, and regeneration of organ systems, including the CNS [4,9,13-14]. However, in disease states, EVs can propagate pathogenic proteins or miRNAs, facilitating the spread and exacerbation of the disease. For instance, in Alzheimer’s disease (AD) and Parkinson’s disease (PD), EVs can propagate pathogenic proteins such as amyloid-beta and alpha-synuclein to perpetuate neuroinflammation and neurodegeneration, resulting in significant brain dysfunction [15-17]. Characterization of the cargo of EVs isolated from body fluids such as the cerebrospinal fluid, blood, saliva has also been found to be beneficial for identifying prognostic and diagnostic markers of various diseases [7]. EVs shed by stem/progenitor cells, on the other hand, carry a cargo of therapeutic miRNAs and proteins, which could protect cell types, reduce oxidative stress and inflammation, increase neurogenesis, and improve cognitive and mood function in disease states [3-7,9,18-19]. Also, large amounts of stem cell-derived EVs could be targeted to neurons and glia in virtually all regions of the brain through intranasal administrations [4,5,10]. Owing to these properties, stem cell-derived EVs or EVs fortified with unique miRNAs, mRNAs, and proteins have received significant interest in promoting tissue repair and regeneration after injury or disease in multiple organ systems. EVs could also be used as vehicles to deliver therapeutic materials to specific target cells in various organs.

In this special issue, experts in their respective fields have provided authoritative reviews, updates, perspectives, or original research findings on the role of EVs in aging and senescence, the utility of evaluating EVs in body fluids for understanding the pathophysiology or progression of various diseases such as PD, Multiple Sclerosis (MS), Chronic Traumatic Encephalopathy (CTE), and Morphine induced amyloidopathy. Also, a series of articles discussed the promise of stem cell-derived EVs for treating PD, MS, Sjogren’s Syndrome, and Inflammatory Bowel Disease, and advancements in loading EVs to deliver nucleic acid therapies.

## Characterization of EVs for understanding the pathophysiology of various diseases

A review on astrocyte-derived EVs (ADEVs) by Rouillard and associates discussed the influence of ADEVs on intercellular communication in physiological and pathological conditions, aging, and senescence [20]. The exciting aspects discussed include the role of ADEVs from activated astrocytes in microglial transformation into antiinflammatory phenotypes after traumatic brain injury, superoxide dismutase 1 (SOD1) mutant astrocytes releasing EVs promoting the activation of microglia, EVs released from IL-1β activated astrocytes promoting peripheral leukocyte recruitment into the brain, and inhibition of oligodendrocyte differentiation by ADEVs released from senescent astrocytes. The authors also conferred the utility of ADEVs as prognostic and diagnostic markers for identifying diseases such as AD and traumatic brain injury (TBI), how the cargo of ADEVs is determined, whether the cargo of ADEVs could be altered in a cell-specific manner, and the therapeutic promise and cellular targets of ADEVs.

Reviews by Upadhya and Shetty, and Leggio and associates, discussed plasma-derived EVs as novel diagnostic and prognostic biomarkers for PD, particularly the measurement of alpha-synuclein (α-syn) in EVs as a possible biomarker for PD [21-22]. The authors also emphasized the role and function of EVs in α-syn-mediated neurodegeneration and α-syn spread from neurons to glia, resulting in the inflammatory response in PD. The other potential EV markers such as phosphorylated Ser(P)-1292, leucine-rich repeat kinase 2 (LRRK2*)*, protein deglycase DJ-1 (PARK*7*), the cellular prion protein (PrPC), apolipoprotein A1 (APO A1), apolipoprotein J (APO J) were also discussed. However, they highlighted that additional studies are needed to validate them as authentic PD markers. Furthermore, Leggio and coauthors propose using mitochondria-derived EVs (MDEVs) as an innovative source of vesicle-carried PD biomarkers [22].

Manu and coauthors discussed EVs as pro- and anti-inflammatory mediators and biomarkers in MS [23]. Notably, the dual role of EVs originated by immune cells in promoting the progression of MS or restraining the progression of MS was discussed. The role of EVs in promoting the progression of MS is evident from the ability of plasma EVs of experimental autoimmune encephalomyelitis (EAE) mice to induce the spontaneous relapse-remitting MS phenotype in myelin oligodendrocyte glycoprotein (MOG) immunized mice, a prototype of MS that typically displays monophasic disease. Furthermore, the fibrinogen in the transferred plasma EVs could drive the CD8+ T cells, resulting in spontaneous EAE in transferred mice. The authors also highlighted that EV miRNA in the plasma samples of MS patients could target regulatory T cells to disrupt the immune regulatory mechanism [23].

CTE, a progressive brain disease, is linked to repetitive head impacts such as concussions in boxers, hockey players, and American football players [24-25]. CTE is a type of tauopathy typified by the deposition of phosphorylated microtubule-associated tau (p-tau) protein. The mechanism of disease spread, especially the propagation of misfolded tau in CTE, is yet to be fully understood. By quantifying total and p-tau and profiling 516 common proteins in brain-derived EVs from CTE and control brain tissue samples via proteomics, Muraoka and colleagues suggested that the combination of tau and cell type-specific molecules from brain cells, including the plexin-A4 (PLXNA4), synaptosomal-associated protein 25 (SNAP-25) or ubiquitin-like modifier activating enzyme 1 (UBA1) may serve as potential monitoring biomarker candidate molecules in CTE patient body fluid samples [24]. In another study, the same research group investigated protein profiles of EVs separated from the plasma of former National Football League (NFL) players at risk for CTE [25]. The authors identified 675 proteins in plasma EVs, of which 17 proteins were significantly differentially expressed between former NFL players and controls. Levels of t-tau and p-tau181 in EVs were also significantly different between the groups. Through additional machine learning analysis, the authors suggested that a combination of collagen type VI alpha 3 and 1 chain (COL6A3 and COL6A1) and reelin could distinguish former NFL players from controls with 85% accuracy [25].

Pinson and colleagues discussed diseases typically associated with aging in light of early life exposures and conferred the potential role of EVs as mediators of lasting consequences [26]. The authors initially focused on prenatal alcohol or nicotine exposure, maternal stress, or early childhood adversity as initial hits for developing premature aging and diseases in adulthood. Next, they conferred the possible role of EVs as mediators and perpetrators of prenatal and early life insult into diseases of aging, with an emphasis on the role of EVs in developmental origins of congenital heart disease, osteoporosis, osteoarthritis, AD, PD, and Cancer [26].

In an original research article, Sil and Colleagues presented evidence supporting the role of ADEVs in morphine-induced amyloidopathy [27]. Their investigation showed that morphine-induced the expression of the transcription factor, hypoxia-inducible factor 1 alpha (HIF-1α), leading to increased activity of beta-site amyloid precursor protein (APP) cleaving enzyme 1 (BACE1) and eventually resulting in the cleavage of the transmembrane protein APP to its toxic amyloid-beta (Aβ) forms [27]. Such changes resulted in the activation of astrocytes leading to the generation of proinflammatory cytokines. Furthermore, their investigation revealed that seeding ADEVs containing toxic isoforms of amyloids into different brain sites caused neurodegeneration and neuroinflammation. The authors suggested that amyloid-mediated neurodegeneration and neuroinflammation likely contribute to cognitive deficits observed in long-term opiate users [27].

## The promise of EVs for treating various diseases

Upadhya and Shetty deliberated the promise of using stem/progenitor cell-derived EVs for treating PD [21]. The authors highlighted that MSC-derived EVs could reduce apoptosis, protect dopaminergic neurons, increase dopamine concentration in the striatum and improve motor function in animal models of PD. Furthermore, macrophage-derived EVs holding the antioxidant catalase could diminish activated microglia in a PD prototype. They also discussed the promise of intravenous administration of rabies virus glycoprotein (RVG)-expressing EVs loaded with siRNA against α-syn for reducing both α-syn mRNA and protein levels in a transgenic mouse expressing the phosphomimetic human S129D α-syn. Such a strategy also diminished dopaminergic neuron loss and alleviated PD symptoms. However, the authors also pointed out several limitations of EV-based therapy for PD. First, the potential mechanisms by which MSC-EVs mediate beneficial effects in PD is unknown because EVs employed were not characterized for their cargo. Second, the efficacy of MSC-derived EVs or EVs loaded with catalase, dopamine, or siRNAs was tested only in the early phase of PD. Furthermore, the authors emphasized that intranasal administration is the most promising route because EVs quickly permeate the entire brain after the non-invasive IN treatment. Repeated intranasal administrations of therapeutic EVs are likely feasible in a doctor’s office without anesthesia. The authors further proposed that innovations in the EV field would promote nasal sprays holding therapeutic EVs for specific neurological conditions in the future [21].

Manu and colleagues discussed the potential of EVs derived from mesenchymal stem cells (MSCs) to protect oligodendrocytes from DNA damage and administration of EVs from MSCs co-cultured with microglia for assisting myelin repair by recruiting oligodendrocyte precursor in demyelinated regions [23]. They concluded that EVs generated from MSCs, microglia, and oligodendrocytes have the promise to treat demyelination lesions or restoring immune tolerance in MS [23]. In an original research article, Kim and coauthors presented the potential mechanisms by which EVs generated from human induced pluripotent stem cell (hiPSC)-derived MSCs (iMSCs) promoted therapeutic benefits in a mouse model of Sjogren’s Syndrome [28]. Since the cargo and therapeutic efficacy of MSC-EVs would depend on donor age, MSC sources, and culture conditions, the authors preferred EVs from hiPSC-derived MSCs (iMSCs) in this study. They reported that EVs from early-passage iMSCs have improved immunomodulatory potency than EVs from late-passage iMSCs in TLR4-stimulated splenocytes and a mouse model of primary Sjögren’s syndrome [28]. Proteomics and microRNA sequencing investigations further demonstrated distinctive molecular profiles of iMSC-EVs with or without immunomodulation capacity. Moreover, manipulation of transforming growth factor-beta 1 (TGF-β1) and miRNAs miR-21 and miR-125b levels in iMSC-EVs considerably affected the immunosuppressive effects iMSC-EVs [28]. The authors concluded that TGF-β1 and miR-21 as key effectors mediating the EV-mediated immunomodulation and proposed miR-125b as a negative regulator [28].

Yu and associates tested the efficacy of EVs generated from human adipose tissue-derived MSCs (hAD-MSCs) for protection against dextran sodium sulfate (DSS)-induced inflammatory bowel disease (IBD) through intestinal-stem-cell and epithelial regeneration [29]. IBD is a severe disease in most patients but has effective treatment currently. In this study, the authors intravenously administered EVs from hAD-MSCs into mice treated with DSS. Their evaluation revealed that hAD-MSC-EVs promoted functional recovery, downregulated inflammatory responses, reduced intestine cell apoptosis, increased epithelial regeneration, and maintained intestinal barrier integrity. Through additional *in vitro* studies using colon organoids, the authors demonstrated that hAD-MSC-EVs can promote the proliferation and regeneration of intestinal stem cells and epithelial cells and ameliorate inflammation in colon organoids treated with TNF-α. The authors concluded that hAD-MSC-EVs have a promise to treat IBD [29].

## Progress in EV engineering

Jurgielewicz and colleagues discussed the current understanding of the biological role of EVs, as well as the advancements in loading EVs to deliver nucleic acid therapies [30]. Specifically, they discussed the current methods and associated challenges in loading EVs and the prospects for utilizing the inherent characteristics of EVs as a delivery vector of nucleic acid therapies for genetic disorders [30]. The current nucleic acid delivery strategies discussed include chemical modifications, nanoparticles, and viral vectors. The authors conferred EVs as natural delivery vectors of nucleic acids and their advantages. They also elaborated on techniques for loading EVs with nucleic acids using pre-isolation approaches such as overexpression using viral or other transfections and post-isolation methods such as electroporation, sonication, chemical transfection, and passive loading [30]. The authors mentioned that EV-based delivery of therapeutic nucleic acids is a promising approach to deliver new precision medicine treatments for various genetic diseases. They highlighted several advantages of using EVs for delivering nucleic acids. First, EVs have an intrinsic ability to protect nucleic acid cargo. Second, EVs can cross physiological barriers, including the blood-brain barrier. Third, EVs are highly stable, have low toxicity and immunogenicity. The authors concluded that the EV-based delivery of nucleic acids has already made significant progress, but further studies are needed to support the preclinical findings to advance nucleic acid-loaded EV therapy to the clinic [30].
